# Age-Dependent Association of Hepatic Steatosis with Vertebral Trabecular Attenuation in Asymptomatic Adults

**DOI:** 10.3390/jcm15218409

**Published:** 2026-10-28

**Authors:** Yoshinao Koike, Tsutomu Endo, Ryo Fujita, Yuichi Hasegawa, Shotaro Fukada, Kiyofumi Komano, Hiroyuki Tachi, Akinobu Nakamura, Yuki Oe, Ken Kadoya, Hideki Sudo, Katsuhisa Yamada, M Alaa Terkawi, Ryota Suzuki, Huohuo Xue, Tomoya Sato, Norimasa Iwasaki

**Affiliations:** 1 Department of Orthopedic Surgery, Hokkaido University Graduate School of Medicine, Sapporo 060-8638, Japan; sunday_yoshi@yahoo.co.jp (Y.K.);; 2 Spine Center, Hakodate Central General Hospital, Hakodate 040-8585, Japan; 3 Department of Rheumatology, Endocrinology and Nephrology, Faculty of Medicine and Graduate School of Medicine, Hokkaido University, Sapporo 060-8638, Japan; 4 Division of Diabetes and Endocrinology, Department of Medicine, NTT Sapporo Medical Center, Sapporo 060-0061, Japan

**Keywords:** hepatic steatosis, Hounsfield unit, vertebral trabecular attenuation, opportunistic screening, younger adults

## Abstract

**Background/Objectives**: Preserving skeletal health before osteoporosis develops has become an important challenge in preventive medicine. In addition to aging, metabolic dysfunction has emerged as a potential contributor to skeletal deterioration. Hepatic steatosis, a manifestation of systemic metabolic dysfunction, has been associated with impaired bone health; however, whether this relationship differs according to age remains unclear, particularly among asymptomatic adults. This study investigated the age-specific association between hepatic steatosis and CT-derived vertebral trabecular attenuation in adults undergoing voluntary health screening. **Methods**: This cross-sectional study included 553 asymptomatic Japanese adults (median age, 51 years; range, 20–88 years) who underwent non-contrast trunk CT during voluntary health checkups. Hepatic steatosis was assessed using the liver-to-spleen Hounsfield unit (HU) ratio (L/S ratio), with lower values indicating greater hepatic fat accumulation. Vertebral trabecular attenuation was assessed using the mean L1–L3 HU value. Participants were categorized as younger (≤49 years), middle-aged (50–64 years), or older (≥65 years). Multivariable regression analyses adjusted for demographic, clinical, and lifestyle factors were performed overall and by age group. An additional model assessed whether the association between the L/S ratio and vertebral HU values varied with age. **Results**: In the overall cohort, a lower L/S ratio was independently associated with lower vertebral HU values (β = 0.088, *p* = 0.018). In age-stratified analyses, this association was most evident in the younger group (β = 0.169, *p* = 0.010), but was attenuated in the middle-aged (β = 0.073, *p* = 0.260) and older groups (β = −0.113, *p* = 0.355). This age-related attenuation was statistically significant (β = −0.112, *p* = 0.004). **Conclusions**: Hepatic steatosis was independently associated with lower CT-derived vertebral trabecular attenuation, with the strongest association observed in adults aged ≤ 49 years. Although unmeasured factors, including menopausal status, may partly explain the observed age-dependent pattern, these findings suggest that hepatic steatosis may be relevant to skeletal health in younger adults and could help identify individuals who may benefit from early assessment and preventive care.

## 1. Introduction

Preserving skeletal health before osteoporosis develops has become an important goal in preventive medicine. In addition to aging and hormonal changes, increasing evidence suggests that systemic metabolic dysfunction contributes to skeletal deterioration [1]. Among metabolic disorders, hepatic steatosis has attracted growing attention because it is increasingly prevalent worldwide, including among younger and middle-aged adults, and is closely linked to systemic metabolic dysfunction [2,3]. Experimental and clinical studies have linked hepatic steatosis to impaired skeletal health, suggesting that chronic inflammation, oxidative stress, and endocrine alterations accompanying hepatic steatosis may adversely affect bone homeostasis [4,5,6,7]. These findings raise the possibility that metabolic dysfunction associated with hepatic steatosis may influence skeletal health before osteoporosis becomes clinically apparent.

Despite growing recognition of the liver–bone relationship, evidence in younger adults remains limited. Previous studies have reported associations between hepatic steatosis and bone mineral density; however, whether this association differs according to age remains unclear [8,9]. In routine clinical practice, dual-energy X-ray absorptiometry (DXA) screening is uncommon in younger adults without specific risk factors [10]. Likewise, hepatic steatosis is often clinically silent and may remain unrecognized in younger adults [11]. Consequently, little is known about whether metabolic dysfunction associated with hepatic steatosis is already accompanied by skeletal alterations before age-related bone loss predominates.

Opportunistic screening using non-contrast computed tomography (CT) offers a practical approach to address this knowledge gap, as it enables the simultaneous assessment of hepatic steatosis and vertebral trabecular attenuation—measured in Hounsfield units (HU)—from routinely acquired images without additional radiation exposure [12,13,14]. The relationship between hepatic steatosis and vertebral trabecular attenuation may differ across the life course because the determinants of skeletal health change with age. While the influence of metabolic dysfunction on the skeleton may be more readily detectable before substantial age-related bone loss develops, age-related structural changes likely dominate vertebral attenuation in older adults. We therefore hypothesized that hepatic steatosis is associated with lower vertebral HU values and that the strength of this association varies according to age.

To test this hypothesis, the present study aimed to investigate age-specific associations between hepatic steatosis and CT-derived vertebral trabecular attenuation in adults undergoing voluntary health screening. By clarifying these age-related differences, we seek to improve the understanding of liver–bone crosstalk over the life course and inform future strategies for the early recognition of skeletal risk.

## 2. Materials and Methods

### 2.1. Study Design

This cross-sectional study was conducted at a health screening institution in a city of approximately 240,000 residents in northern Japan between April 2020 and March 2021. The study was approved by the institutional ethics committee (approval number: 2020–14; date of approval: 19 September 2025), and the requirement for informed consent was waived because of the retrospective nature of the study and the use of deidentified data.

### 2.2. Participants

This study included a cohort of 12,740 Japanese individuals who underwent health screening examinations. Most participants were local residents, employees of local businesses, and hospital staff. Participation in trunk CT was voluntary, with participants selecting the scan area (neck-to-chest, abdomen-to-pelvis, or neck-to-pelvis) based on personal preference, primarily for screening of thoracic and abdominal malignancies, as well as other conditions detectable by CT. The CT examination was not part of a national or local government screening program. Of these, 654 participants who had chest and abdominal CT scans were selected for further analysis. Participants with prior osteoporosis treatment, vertebral lesions or artifacts that hindered HU assessment, or multiple missing values in the variables assessed in this study were excluded, leaving 553 participants for the final analysis.

### 2.3. Background Data and Comorbidities

Age, sex, and body mass index (BMI) were obtained from the health examination data of individuals. Furthermore, to assess comorbidities, data from a comprehensive questionnaire were combined with other sources to maximize information. This questionnaire was not newly developed for the current research but is rather the standard health checkup questionnaire routinely administered at our institution (Supplementary Material). We focused on previously reported factors associated with bone metabolism [15,16,17,18]. Individuals were classified as having diabetes mellitus if they had a history of diabetes medication use or a glycated hemoglobin level ≥ 6.5% (National Glycohemoglobin Standardization Program). Individuals with a prior diagnosis of emphysema or radiologist-confirmed emphysema on chest CT were classified as having the condition. Participants currently or previously treated for collagen diseases, malignant tumors, or thyroid dysfunction (hyperthyroidism or hypothyroidism), as indicated by the questionnaire, were classified accordingly. However, chronic kidney disease (CKD) classification was based on an estimated glomerular filtration rate ≤ 60 mL/min/1.73 m^2^ during physical examination, as questionnaire data were not used. Additionally, information on daily alcohol consumption and smoking history was obtained from the questionnaire.

### 2.4. Hounsfield Unit Measurement of the Vertebral Body Using Computed Tomography

**Figure 1:**
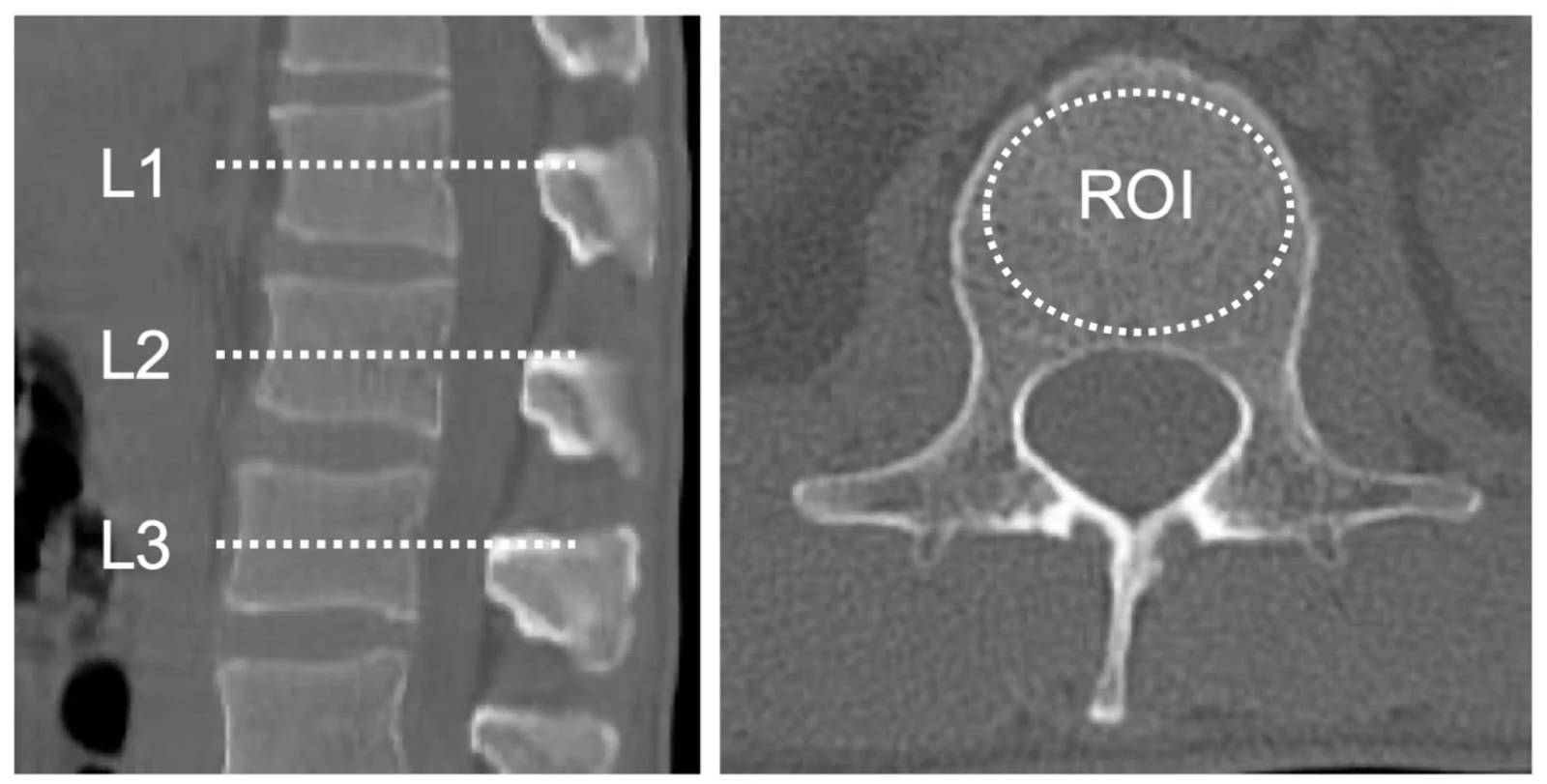
Measurement of vertebral HU using CT. ROIs were placed on axial images at the mid-body of the L1–L3 vertebrae. The HU value of each vertebral ROI was measured, and the mean L1–L3 HU value was used for analysis. HU, Hounsfield unit; CT, computed tomography; ROIs, regions of interest.

Axial slices from spine CT images were used to assess the vertebral body. All CT images were obtained using the Aquilion™ ONE/GENESIS Edition system (Canon Medical Systems Corporation, Otawara, Japan) with a tube voltage of 120 kVp. Regions of interest (ROIs) were placed in the cancellous bone of axial cross-sectional images on the central vertebral body to measure HU values, ensuring maximal inclusion of cancellous bone while avoiding cortical bone [13,19]. HU values were measured at the L1–L3 vertebrae. CT-derived trabecular attenuation correlates with bone mineral density and bone strength and is widely used for opportunistic skeletal assessment [13,14]. The mean L1–L3 HU value was used as the primary measure of vertebral trabecular attenuation in this study (Figure 1). Two spine surgeons performed the measurements. Interobserver reliability was assessed in a subset of 40 participants independently evaluated by both surgeons using intraclass correlation coefficients (ICCs) based on a two-way random-effects model for absolute agreement (Table A1).

### 2.5. Assessment of Hepatic Steatosis

**Figure 2:**
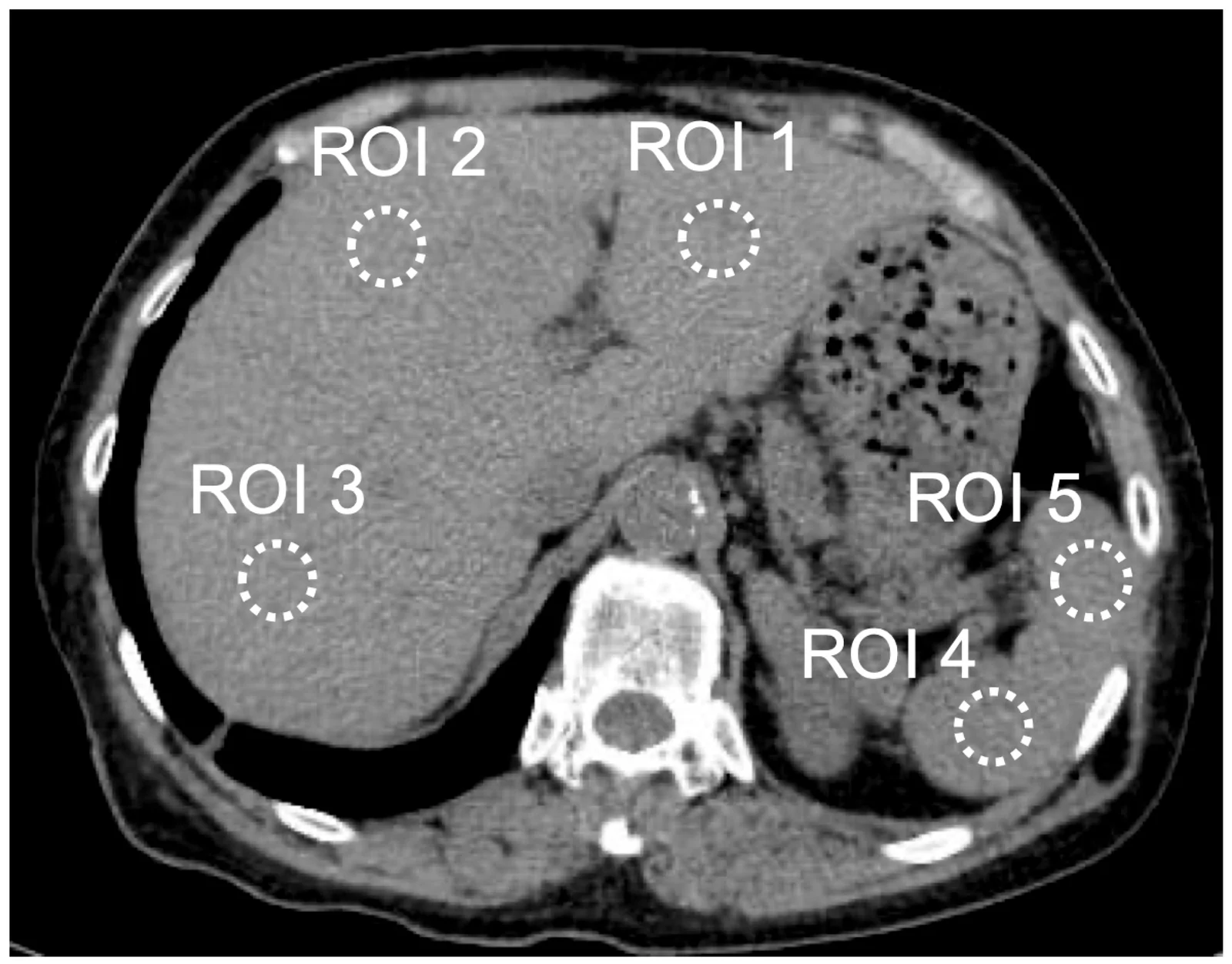
Measurement of the L/S ratio. Two ROIs were placed in the right liver lobe, one in the left liver lobe, and two in the spleen. L/S ratio, liver-to-spleen Hounsfield unit ratio; ROIs, regions of interest.

Hepatic steatosis severity was assessed using the liver-to-spleen HU ratio (L/S ratio) [20,21,22]. HU values for the liver and spleen were obtained using 400 mm^2^ ROIs. Two ROIs were placed in the right liver lobe, one in the left lobe, and two in the spleen, excluding hepatic vessels and artifacts to ensure uniform parenchymal attenuation (Figure 2). The L/S ratio was calculated using the average HU values of each liver and spleen segment. Liver fat accumulation was evaluated according to the guidelines of the Japanese Society of Gastroenterology, where L/S ratios of 0.9, 1.01, 1.12, and 1.2 indicate ≥60%, 30–60%, ≤30%, and no fat deposition, respectively [23]. Therefore, a lower L/S ratio indicates greater liver fat accumulation. All measurements were performed by a radiologist.

### 2.6. Assessment of Liver Fibrosis

The fibrosis index based on four factors (FIB-4 index) is a noninvasive tool originally developed for estimating liver fibrosis severity in patients with chronic liver disease [24]. Although its diagnostic accuracy in general health-screening cohorts—particularly among younger asymptomatic individuals—has been debated, it remains a widely accepted practical surrogate in population-based studies. The FIB-4 index was calculated using the following equation: FIB-4 = [age (years) × aspartate aminotransferase (U/L)]/[platelet count (10^9^/L) × √(alanine aminotransferase (U/L))]. Recently proposed cutoff FIB-4 index values were referenced for this study: low and high cutoffs were 1.05 and 1.21, 1.24 and 1.96, 1.88 and 2.67, and 1.95 and 2.67 for individuals aged ≤49 years, 50–59 years, 60–69 years, and ≥70 years, respectively [24]. In this study, individuals above the high cutoff value were classified as having liver fibrosis, and those below as not having it.

### 2.7. Classification of Participants by Age Group

Participants were categorized into three age groups—≤49 years (younger age), 50–64 years (middle age), and ≥65 years (older age)—and their background data, vertebral HU values, liver-related metrics, and comorbidities were compared across groups. These age categories were predefined to represent younger adulthood before substantial age-related skeletal decline (≤49 years), transitional middle age (50–64 years), and older adulthood (≥65 years).

### 2.8. Assessment of Factors Associated with Vertebral Hounsfield Unit Value Using Multivariable Linear Regression Analysis

Multivariable linear regression analysis was conducted to identify factors affecting vertebral HU values. The mean HU of L1–L3 served as the response variable. Explanatory variables included the L/S ratio, age, sex, BMI, FIB-4 index, comorbidities (diabetes, CKD, collagen disease, emphysema, malignant tumor, and thyroid dysfunction), alcohol consumption, and smoking history. Additionally, multivariable linear regression analyses were performed within each age group to examine differences in the effect of hepatic steatosis severity on vertebral HU values. The same covariates were included in the overall and age-stratified multivariable regression models. In the age-by-sex subgroup analyses, collagen disease, emphysema, and thyroid dysfunction were excluded from the covariates because some subgroups had no participants with these conditions. All continuous variables were standardized before analysis.

As a sensitivity analysis, the overall and age-stratified multivariable analyses were repeated using L1 HU values instead of mean L1–L3 HU values.

### 2.9. Clinically Interpretable Secondary Analysis Using Binary Hepatic Steatosis Definitions

To facilitate clinical interpretation, additional multivariable regression analyses were performed by replacing the continuous L/S ratio with a binary hepatic steatosis variable. In the primary definition, hepatic steatosis was defined as an L/S ratio < 1.1, based on previous non-contrast CT studies using liver-to-spleen attenuation measures to assess hepatic steatosis [20]. The mean HU of L1–L3 served as the response variable. Explanatory variables included binary hepatic steatosis, age, sex, BMI, FIB-4 index, comorbidities, alcohol consumption, and smoking history.

In contrast to the main analyses, continuous variables were entered into these models without standardization so that the regression coefficients could be interpreted in their original clinical units.

Sensitivity analyses were also performed using a more stringent L/S ratio threshold of <1.0, which has been reported to have high specificity for detecting at least moderate hepatic steatosis on non-contrast CT [25].

### 2.10. Interaction Analysis for the Association Between Hepatic Steatosis and Vertebral Hounsfield Unit Values

To formally support the age-dependent pattern observed in the age-stratified analyses, an L/S ratio × age interaction term was added to the multivariable linear regression model. Continuous variables were standardized before analysis. The model included the same covariates as the primary multivariable analysis.

This analysis was also repeated using L1 HU values as a sensitivity analysis.

### 2.11. Missing Data

Missing data included the FIB-4 index (0.9%, 5/553) and estimated glomerular filtration rate levels for CKD evaluation (2.0%, 11/553). For group comparisons, missing data were left blank. All multivariable regression analyses were performed using complete-case data. The overall multivariable analysis included 537 participants, comprising 228 younger, 240 middle-aged, and 69 older adults. In the age-by-sex subgroup analyses, the numbers of male and female participants were 122 and 106 in the younger group, 131 and 109 in the middle-aged group, and 56 and 13 in the older group, respectively. The same complete-case dataset was used for all sensitivity analyses.

### 2.12. Statistical Analysis

Data were analyzed using R (version 4.5.1; R Foundation for Statistical Computing, Vienna, Austria). Fisher’s exact test was used to compare binary variables between groups using the fisher.test function. Continuous variables were compared using the Wilcoxon rank-sum test implemented with the wilcox_test function in the coin package (version 1.4.3). Correlations between the L/S ratio and mean vertebral HU values were assessed using Pearson’s correlation coefficient with the cor.test function in the overall population and in subgroups defined by sex and age group. Multivariable linear regression analyses were performed using the lm function. Multicollinearity among the explanatory variables was assessed by calculating variance inflation factors (VIFs) using the vif function in the car package (version 3.1.3). In the main multivariable regression model, all VIF values were below 1.23. Across the subgroup, secondary, and sensitivity analyses, the maximum VIF was 4.96, while the maximum VIF for the L/S ratio was 2.88. Interobserver reliability was assessed using the ICC function in the psych package (version 2.5.6).

The overall multivariable model using the continuous L/S ratio was the primary analysis. Age-stratified analyses were performed to examine the association across age groups, and an additional model including an L/S ratio × age interaction term was used to assess whether the association varied with age. Pearson correlation analyses were performed to examine unadjusted relationships across age and sex groups. The analysis using an L/S ratio < 1.1 was performed as a secondary analysis, while analyses using an L/S ratio < 1.0 and L1 HU values alone were performed as sensitivity analyses. Bonferroni correction was applied to the multiple Pearson correlation analyses. No uniform multiplicity correction was applied across the regression-based analyses because these analyses addressed different aspects of the study question or assessed the robustness of the main findings and were not treated as a single family of parallel hypothesis tests.

All statistical tests were two-sided, and *p* < 0.05 was considered statistically significant unless otherwise specified.

### 2.13. Use of Artificial Intelligence Tools

ChatGPT (GPT-5.6; OpenAI, San Francisco, CA, USA) and Gemini (Gemini 2.5 Pro; Google, Mountain View, CA, USA) were used to assist with language editing and to improve the clarity and presentation of the manuscript during manuscript preparation and revision. No AI tools were used for study design, data collection, statistical analysis, or generation of the study results. All AI-assisted suggestions were critically reviewed and revised by the authors.

## 3. Results

### 3.1. Differences in the Health Status of Individuals Between Age Groups

In the participant characteristics shown in Table 1, mean vertebral HU (average L1–L3) decreased stepwise with age: the median was 190.3 HU in the younger group (≤49 years), 145.2 HU in the middle-aged group (50–64 years), and 130.7 HU in the older group (≥65 years). By contrast, the L/S ratio and BMI did not differ substantially between age groups. The FIB-4 index, a simple surrogate for liver fibrosis, increased significantly with age (median: 0.80 in the younger group, 1.19 in the middle-aged group, and 1.62 in the older group), and a larger proportion of participants in the middle-aged and older groups exceeded the age-adjusted cutoffs (Table 1).

Regarding comorbidities, diabetes and CKD were significantly more prevalent in the older age group, whereas smoking history did not differ significantly across age groups (Table 1).

### 3.2. Correlation Between the L/S Ratio and Vertebral HU Values

**Figure 3:**
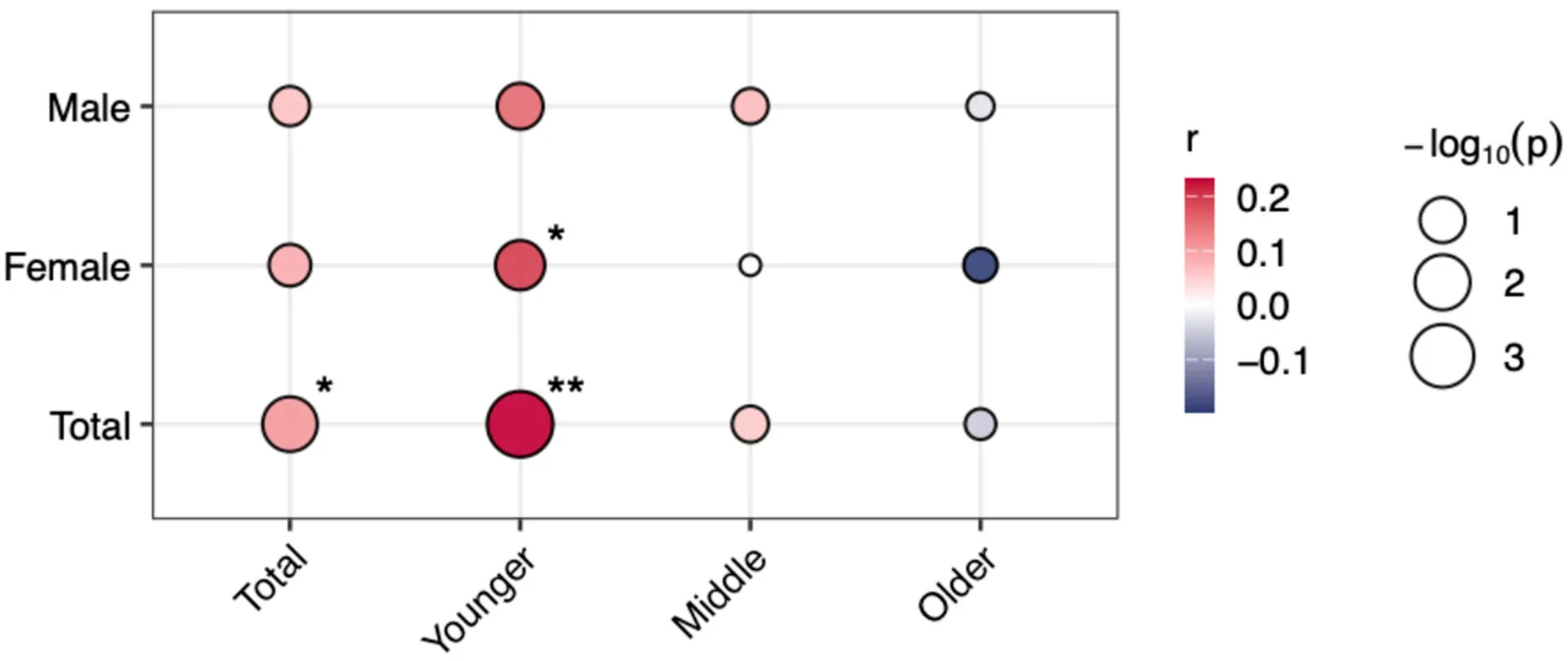
Correlation between the L/S ratio and vertebral HU values. Plots show the correlation between mean vertebral HU values and the liver-to-spleen (L/S) ratio according to sex (rows) and age group (columns). The Total row includes both men and women, and the Total column includes all age groups combined. The direction and magnitude of Pearson’s correlation coefficients (r) are represented by circle color, whereas statistical significance is represented by circle size, with larger circles indicating smaller *p* values. * *p* < 0.05; ** *p* < 0.05/12, corresponding to the Bonferroni-corrected significance threshold for 12 comparisons. HU, Hounsfield unit; L/S ratio, liver-to-spleen Hounsfield unit ratio.

We examined simple correlations to assess the unadjusted relationship between hepatic fat (L/S ratio) and vertebral trabecular attenuation within each sex and age group before multivariable adjustment. A significant correlation that remained after Bonferroni correction was observed in the younger group combining both men and women (r = 0.232, 95% Confidence Interval [CI]: 0.107 to 0.350; *p* < 0.001) (Figure 3). Because lower L/S ratios indicate greater hepatic fat accumulation, the positive correlation reflects an association between greater hepatic steatosis and lower vertebral HU values. In addition, nominally significant correlations were observed in the overall population combining both sexes and all age groups (r = 0.108, 95% CI: 0.025 to 0.190; *p* = 0.011) and in younger women (r = 0.197, 95% CI: 0.009 to 0.371; *p* = 0.040), although neither met the Bonferroni-corrected significance threshold.

### 3.3. Association Between Hepatic Steatosis and Vertebral Hounsfield Unit Values

Table 2 shows the multivariable linear regression analysis results of factors affecting vertebral HU values. Increased age was most significantly associated with lower vertebral HU values (β = −0.530, 95% CI: −0.604 to −0.457; *p* < 0.001). The L/S ratio was positively associated with vertebral HU values (β = 0.088, 95% CI: 0.015–0.160; *p* = 0.018), indicating that greater hepatic steatosis was independently associated with lower vertebral HU values. Furthermore, male sex (β = −0.179, 95% CI: −0.334 to −0.023; *p* = 0.024) and smoking history (β = −0.182, 95% CI: −0.332 to −0.032; *p* = 0.017) were associated with lower vertebral HU values (Table 2).

### 3.4. Age-Dependent Association Between Hepatic Steatosis and Vertebral HU Values

**Figure 4:**
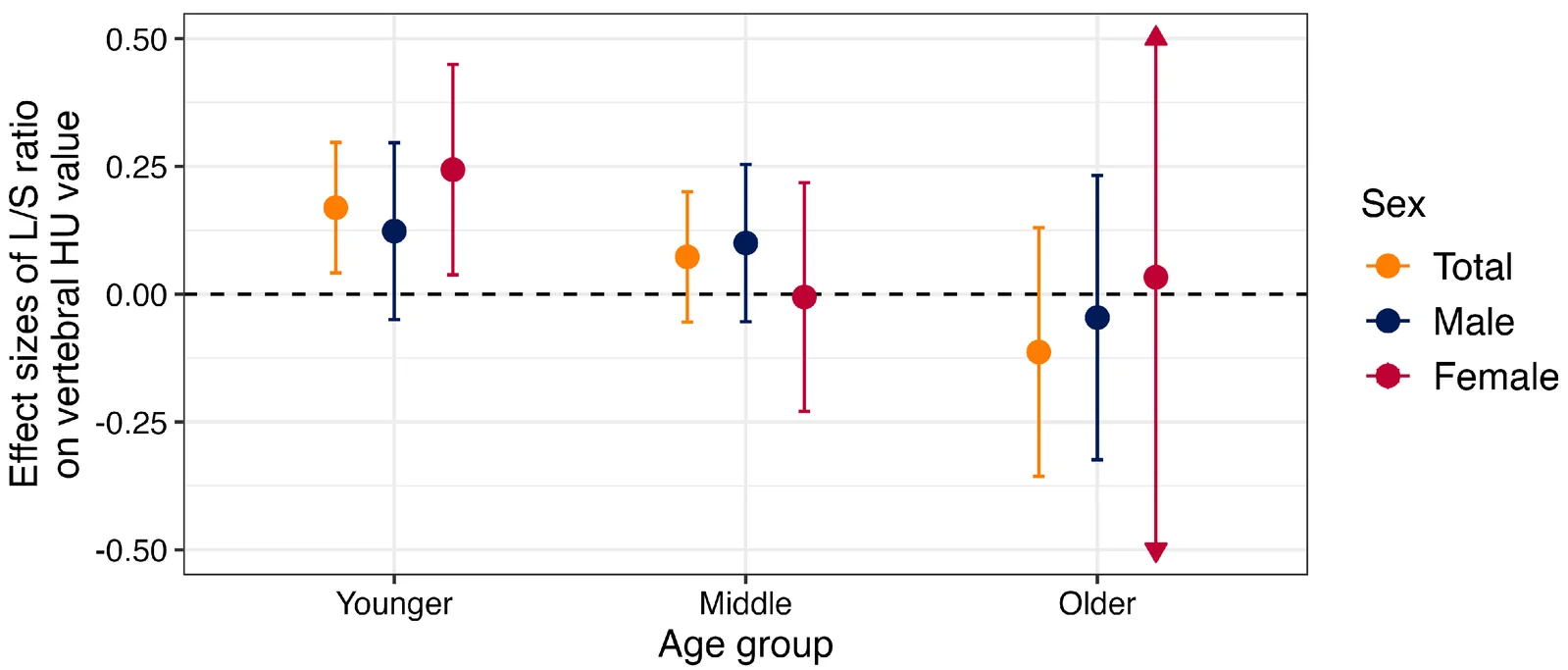
Effect size of the L/S ratio on vertebral HU values across age groups. Error bars indicate 95%CIs. Arrows indicate that the 95% CIs extend beyond the displayed y-axis range. L/S ratio, liver-to-spleen Hounsfield unit ratio; HU, Hounsfield unit; CI, confidence intervals.

To examine whether the association between hepatic steatosis and vertebral HU values differed by age, we performed age-stratified multivariable analyses. The association between the L/S ratio and vertebral HU values was strongest in the younger group (β = 0.169, 95% CI: 0.041 to 0.297; *p* = 0.010), whereas no statistically significant association was observed in the middle-aged group (β = 0.073, 95% CI: −0.054 to 0.200; *p* = 0.260) or the older group (β = −0.113, 95% CI: −0.356 to 0.130; *p* = 0.355). A similar age-dependent pattern was observed in both men and women (Figure 4).

To formally evaluate the age-dependent pattern observed in the stratified analyses, we performed an additional interaction analysis. A significant interaction was observed between the L/S ratio and age (β = −0.112, 95% CI: −0.188 to −0.036; *p* = 0.004), indicating that the positive association between the L/S ratio and vertebral HU values weakened with increasing age (Table A2). In sex-stratified analyses, the interaction estimates were similar in direction and magnitude in men (β = −0.080) and women (β = −0.117), although neither reached statistical significance (Table A2).

Sensitivity analyses using L1 HU values alone yielded results consistent with those using mean L1–L3 HU values, including the overall association, age-stratified pattern, and L/S ratio × age interaction (Table A3).

### 3.5. Age-Dependent Differences in Vertebral HU Values Associated with Binary Hepatic Steatosis

**Figure 5:**
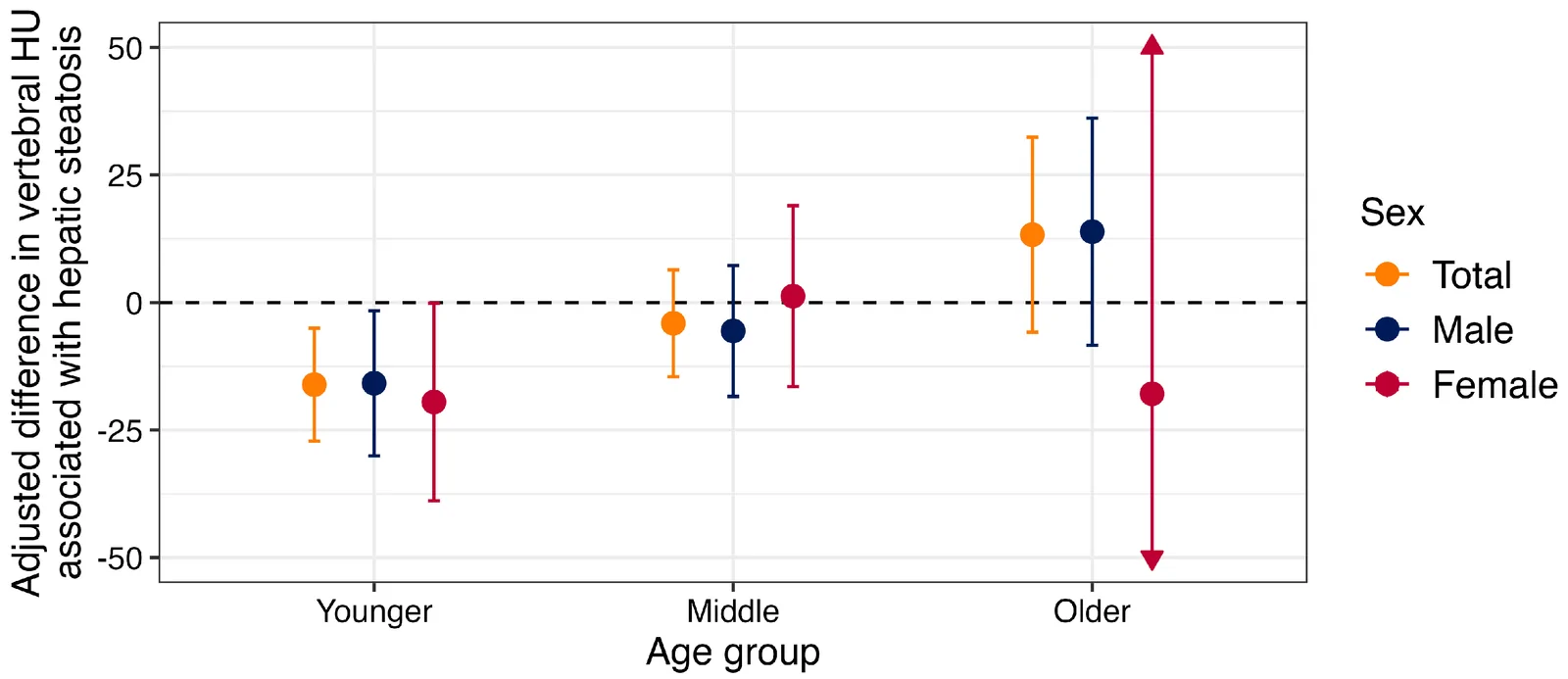
Adjusted difference in vertebral HU values associated with hepatic steatosis across age groups. Values represent beta coefficients for hepatic steatosis from multivariable linear regression models. Error bars indicate 95% CIs. Arrows indicate that the 95% CIs extend beyond the displayed y-axis range. Hepatic steatosis was defined as an L/S ratio < 1.1. L/S ratio, liver-to-spleen Hounsfield unit ratio; HU, Hounsfield unit; CI, confidence interval.

To facilitate clinical interpretation, we repeated the age-stratified multivariable analyses using binary definitions of hepatic steatosis instead of the continuous L/S ratio. When hepatic steatosis was defined as an L/S ratio < 1.1, younger adults with hepatic steatosis had significantly lower vertebral HU values (adjusted difference, −16.093 HU; 95% CI: −27.161 to −5.026; *p* = 0.005), whereas the corresponding differences were smaller and not statistically significant in the middle-aged group (−4.068 HU; 95% CI: −14.546 to 6.410; *p* = 0.445) and older group (13.293 HU; 95% CI: −5.813 to 32.400; *p* = 0.169). A broadly similar age-dependent pattern was observed in both men and women, although the confidence intervals were wider in the sex-stratified analyses (Figure 5). These findings indicate that the age-dependent association was preserved even when hepatic steatosis was defined using a clinically applicable diagnostic threshold.

**Figure A1:**
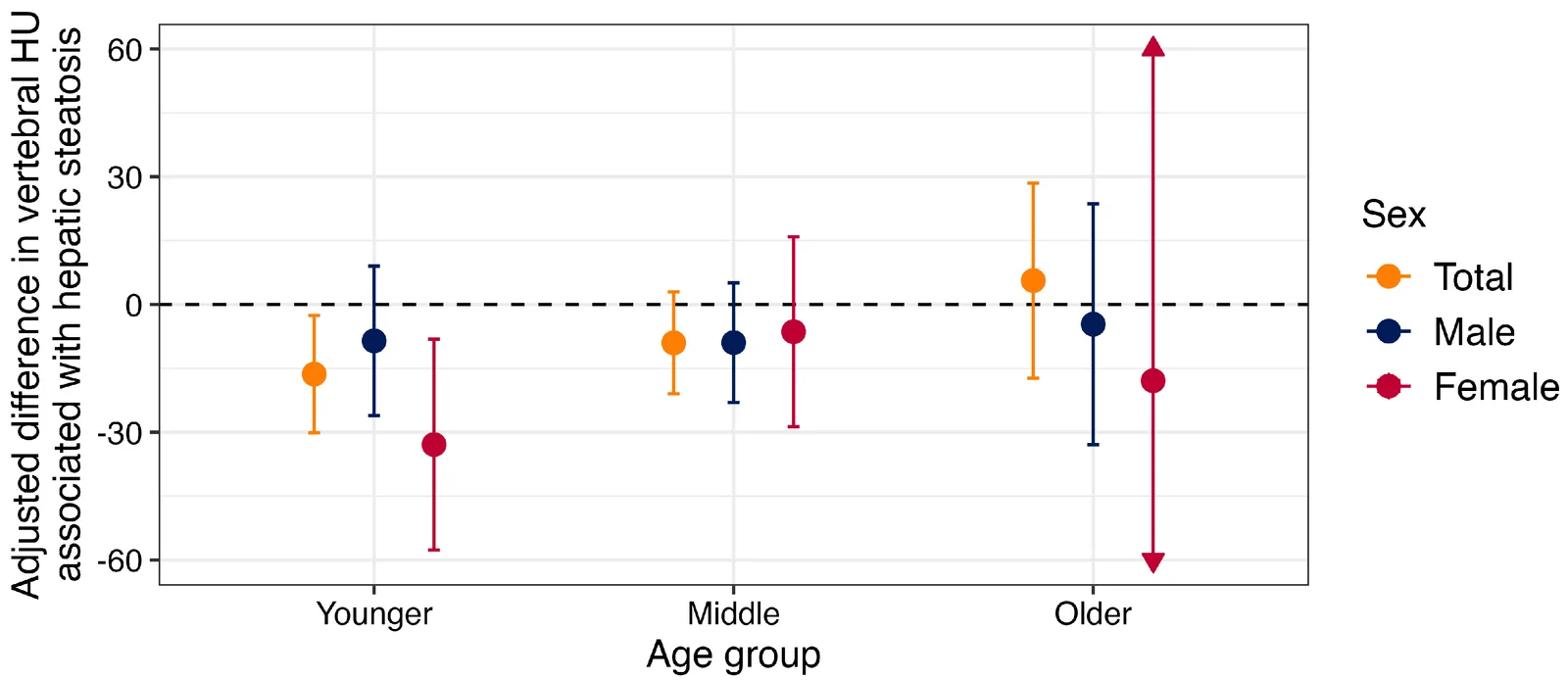
Adjusted difference in vertebral HU values associated with hepatic steatosis across age groups in the sensitivity analysis. Values represent beta coefficients for hepatic steatosis from multivariable linear regression models. Error bars indicate 95% CIs. Arrows indicate that the 95% CIs extend beyond the displayed y-axis range. Hepatic steatosis was defined as an L/S ratio < 1.0. L/S ratio, liver-to-spleen Hounsfield unit ratio; HU, Hounsfield unit; CI, confidence interval.

Sensitivity analysis using the more conservative definition of hepatic steatosis, an L/S ratio < 1.0, showed a similar pattern, with the largest adjusted reduction in vertebral HU values observed in the younger group. This tendency was also observed in both men and women (Figure A1).

## 4. Discussion

This study showed that hepatic attenuation on non-contrast CT, quantified using the L/S ratio, was independently associated with vertebral HU values in a generally healthy population undergoing routine health screening. Notably, this relationship showed an age-dependent pattern.

The principal finding of this study was that the association between the L/S ratio and vertebral HU values differed according to age. A significant positive association was observed in younger adults, whereas the association was attenuated in middle-aged and older adults. The significant L/S ratio × age interaction further supported this age-dependent pattern, and analyses using binary definitions of hepatic steatosis yielded findings consistent with the primary analyses. In younger adults, hepatic steatosis defined as an L/S ratio < 1.1 was associated with approximately 16-HU lower vertebral attenuation after multivariable adjustment; however, the clinical significance of this difference remains uncertain because established HU thresholds for younger adults are lacking. These results suggest that the association between hepatic lipid accumulation and vertebral trabecular attenuation may be more readily detectable in younger adults, before the cumulative effects of aging and other skeletal determinants become more prominent. Because hepatic steatosis is increasingly recognized as a manifestation of systemic metabolic dysfunction rather than an isolated liver disorder [2], its skeletal correlates may be less readily distinguishable in middle-aged and older adults, in whom age-related bone loss and other cumulative influences become more dominant. However, unmeasured menopausal status may also provide an alternative explanation for the attenuated association observed in middle-aged adults, as menopause-related bone loss could influence vertebral HU values independently of hepatic steatosis. Moreover, hepatic steatosis is often asymptomatic [11], and skeletal assessment is uncommon in younger adults [10]; therefore, such skeletal correlates may otherwise remain unrecognized. Because hepatic steatosis is potentially modifiable through lifestyle intervention [26,27], early recognition of this condition may be particularly relevant in younger adults.

A methodological strength of this study was the simultaneous assessment of hepatic and vertebral attenuation on the same non-contrast CT images. Although DXA remains the standard method for evaluating bone mineral density, lumbar measurements can be influenced by degenerative spinal changes, particularly in older adults [19,28]. Vertebral HU measurements are less affected by these changes and provide an objective estimate of trabecular attenuation [19,28]. CT-based hepatic attenuation is also objective and reproducible and is less operator-dependent than ultrasonography [29,30]. Therefore, when abdominal non-contrast CT images are already available, opportunistic assessment of hepatic and vertebral attenuation may provide clinically relevant information even in younger adults without additional imaging or radiation exposure.

Our findings are broadly consistent with previous studies reporting an inverse association between hepatic steatosis and lumbar spine BMD in East Asian populations [8,9]. Lee et al. retrospectively analyzed 3739 postmenopausal women, including 605 with nonalcoholic fatty liver disease, and found that NAFLD was significantly associated with lower lumbar BMD [9]. Similarly, Xia et al. reported an inverse association between ultrasonographically assessed hepatic steatosis and lumbar BMD in Chinese men and postmenopausal women [8]. In contrast, Bhatt et al. found significantly higher vertebral BMD in 162 Indian patients with NAFLD than in 173 age- and sex-matched controls [31]. These inconsistent findings may reflect differences in age, sex, body composition, assessment methods, metabolic confounding, and ethnicity. East Asian populations tend to develop obesity-related metabolic complications at lower BMI levels than Western populations [32,33], which may partly contribute to population differences in the skeletal correlates of hepatic steatosis. Although most previous studies focused on postmenopausal women or middle-aged and older adults, Xie et al. included adults aged 20–59 years and suggested a possible inverse association between NAFLD and lumbar BMD in younger adults [34]. However, whether the relationship between hepatic steatosis and skeletal status changes systematically across adulthood has remained unclear. By including adults across a broad age range, our study extends previous findings by directly evaluating age-dependent differences in this association.

Although higher BMI is generally associated with greater mechanical loading and higher bone mineral density [35], BMI was not significantly associated with vertebral HU values in our study. This finding suggests that the relationship between adiposity and vertebral trabecular attenuation may not be explained by mechanical loading alone. Adipokines such as leptin have been proposed to exert both anabolic and inhibitory effects on bone through peripheral and central pathways [36,37], highlighting the complex relationship between adiposity and skeletal health.

The associations of older age and smoking with lower vertebral HU values are consistent with their established adverse effects on skeletal health. Advancing age is a major determinant of declining vertebral trabecular attenuation [14,38,39], consistent with the strong inverse association between age and vertebral HU values observed in our study. Smoking may impair bone metabolism through alterations in calciotropic hormones and suppression of bone formation [40]. Male sex was also associated with lower vertebral HU values. Previous CT-based studies have reported higher vertebral HU values in women than in men, particularly at younger and premenopausal ages, with the sex difference diminishing with advancing age [14,38,39], broadly consistent with our findings.

It is also important to consider what CT-derived HU values represent in the context of metabolic dysfunction. Although vertebral HU values are widely used as a surrogate for bone status, trabecular attenuation reflects both mineralized bone and bone marrow composition [41]. Hepatic steatosis has also been associated with higher lumbar vertebral marrow fat on MRI [42]. Because greater marrow fat content can reduce CT attenuation [41], the lower vertebral HU values associated with hepatic steatosis in our study may reflect skeletal alterations involving both mineralized bone and marrow composition. From this perspective, CT-derived vertebral attenuation may provide an integrated measure of skeletal changes associated with metabolic dysfunction, rather than a measure of bone mineral density alone.

This study has several limitations. First, its cross-sectional design limits causal inference regarding the relationship between hepatic steatosis and vertebral trabecular attenuation; our findings may reflect a shared metabolic pathogenesis rather than a direct causal pathway. Second, we could not adjust for several important unmeasured confounders, most notably menopausal status. Given that estrogen deficiency is a major determinant of bone loss, the lack of this information may have confounded the attenuated association observed in the middle-aged and older groups. Furthermore, data on vitamin D levels, physical activity, and prior corticosteroid use were unavailable, and reliance on self-reported questionnaires for some comorbidities may be incomplete. Therefore, the observed age-dependent pattern should be interpreted cautiously, as residual confounding by menopausal status and other unmeasured bone-related factors cannot be excluded. Third, the older group had a relatively small sample size, resulting in less precise estimates with wider confidence intervals, and included a higher proportion of men than the younger and middle-aged groups. Accordingly, the lack of a statistically significant association in the older group may partly reflect limited statistical precision and should not be interpreted as evidence of no association. Fourth, although CT-derived HU values are widely accepted surrogate markers of bone mineral density, they do not fully capture all determinants of skeletal strength, including trabecular microarchitecture and bone turnover. Finally, participants were drawn from individuals voluntarily opting for trunk CT during health screenings. This may introduce a selection bias toward health-conscious individuals or those with specific health concerns, potentially limiting the generalizability of our findings to the broader asymptomatic population. However, utilizing retrospective non-contrast CT scans without additional radiation exposure highlights the practicality of this opportunistic, dual-purpose screening for both metabolic and skeletal health.

In conclusion, hepatic steatosis was independently associated with lower vertebral HU values, and this association was most evident in younger adults. These findings suggest that hepatic steatosis in younger individuals may have skeletal correlates and should not necessarily be regarded as an isolated liver finding. When abdominal non-contrast CT images are already available, concurrent assessment of hepatic and vertebral attenuation may provide additional information without further imaging or radiation exposure.

## Figures and Tables

**Table 1:** Background of the participants.

Item	Total (*n* = 553)	Younger (*n* = 233)	Middle Age (*n* = 248)	Older (*n* = 72)
Age	51 (20–88)	44 (20–49)	56 (50–64) *	68 (65–88) †#
Male sex	57.3% (317/553)	53.2% (124/233)	54.0% (134/248)	81.9% (59/72) †#
BMI	23.8 (13.8–44.2)	23.4 (13.8–44.2)	24.0 (15.6–39.8)	23.9 (19.7–31.1)
Liver items				
L/S ratio	1.21 (0.30–1.63)	1.21 (0.30–1.58)	1.21 (0.35–1.63)	1.21 (0.35–1.50)
FIB-4 index	0.99 (0.35–4.74)	0.80 (0.35–1.77)	1.19 (0.41–3.81) *	1.62 (0.72–4.74) †#
>Low cutoff	24.1% (132/548)	13.7% (32/233)	32.2% (79/245) *	30.0% (21/70) #
>High cutoff	4.6% (25/548)	6.4% (15/233)	1.6% (4/245) *	8.6% (6/70) †
Vertebral HU value				
L1	168 (64–327)	194 (75–327)	155 (64–260) *	136.5 (68–216) †#
L2	165 (55–320)	188 (86–320)	147 (61–271) *	132.5 (55–238) †#
L3	154 (46–309)	187 (75–309)	139 (59–273) *	130 (46–209) †#
Mean of L1–L3	161.7 (63.7–318.7)	190.3 (78.7–318.7)	145.2 (69.0–265.7) *	130.7 (63.7–207.7) †#
Coexisting disease				
Diabetes mellitus	8.7% (48/553)	3.9% (9/233)	9.3% (23/248) *	22.2% (16/72) †#
Collagen disease	2.0% (11/553)	1.3% (3/233)	2.8% (7/248)	1.4% (1/72)
Emphysema	4.7% (26/553)	1.7% (4/233)	7.7% (19/248) *	4.2% (3/72)
Cancer	3.8% (21/553)	3.4% (8/233)	2.4% (6/248)	9.7% (7/72) †
Thyroid dysfunction	1.6% (9/553)	1.7% (4/233)	1.2% (3/248)	2.8% (2/72)
CKD	6.5% (35/542)	2.2% (5/228)	7.4% (18/243) *	16.9% (12/71) †#
Daily alcohol consumption	24.1% (133/553)	23.2% (54/233)	22.2% (55/248)	33.3% (24/72)
History of smoking	55.7% (308/553)	52.4% (122/233)	56.5% (140/248)	63.9% (46/72)

*: *p* < 0.05, younger vs. middle-aged group. †: *p* < 0.05, middle-aged vs. older group. #: *p* < 0.05, younger vs. older group. BMI, body mass index; L/S ratio, liver-to-spleen Hounsfield unit ratio; FIB-4 index, fibrosis index based on four factors; HU, Hounsfield unit; L, lumbar; CKD, chronic kidney disease.

**Table 2:** Results of multivariable linear regression analysis.

	β [95% CI]	*p* Value
Age	−0.530 [−0.604, −0.457]	<0.001
Male sex	−0.179 [−0.334, −0.023]	0.024
BMI	0.024 [−0.052, 0.099]	0.540
L/S ratio	0.088 [0.015, 0.160]	0.018
FIB-4 index above high cutoff	−0.024 [−0.357, 0.309]	0.887
Daily alcohol consumption	−0.032 [−0.201, 0.136]	0.706
History of smoking	−0.182 [−0.332, −0.032]	0.017
Diabetes mellitus	−0.096 [−0.355, 0.163]	0.466
Collagen disease	0.112 [−0.409, 0.633]	0.673
Emphysema	−0.093 [−0.435, 0.249]	0.593
Cancer	−0.121 [−0.495, 0.253]	0.525
Thyroid dysfunction	0.514 [−0.038, 1.066]	0.068
CKD	0.085 [−0.204, 0.374]	0.565

CI, confidence interval; BMI, body mass index; L/S ratio, liver-to-spleen Hounsfield unit ratio; FIB-4 index, fibrosis index based on four factors; CKD, chronic kidney disease.

## Data Availability

The datasets used and/or analyzed during the current study are available from the corresponding author on reasonable request.

## Supplementary Materials

The following supporting information can be downloaded at: https://www.mdpi.com/article/10.3390/jcm15218409/s1.

## Abbreviations

The following abbreviations are used in this manuscript:

BMI	Body mass index
BMD	Bone mineral density
CI	Confidence interval
CKD	Chronic kidney disease
CT	Computed tomography
DXA	Dual-energy X-ray absorptiometry
FIB-4	Fibrosis index based on four factors
HU	Hounsfield unit
L/S ratio	Liver-to-spleen Hounsfield unit ratio
MRI	Magnetic resonance imaging
NAFLD	Nonalcoholic fatty liver disease
ROI	Region of interest

## Appendix A

**Table A1:** Interobserver reliability of vertebral HU measurements.

Vertebral Measurement	ICC [95% CI]
L1	0.963 [0.932, 0.980]
L2	0.940 [0.878, 0.969]
L3	0.927 [0.857, 0.962]
Mean L1–L3	0.971 [0.936, 0.986]

ICCs were calculated using a two-way random-effects model for absolute agreement based on single measurements. ICC, intraclass correlation coefficient; CI, confidence interval; HU, Hounsfield unit.

**Table A2:** Multivariable analysis of the interaction between the L/S ratio and age for vertebral Hounsfield unit values.

Population	β [95% CI]	*p* Value
Total	−0.112 [−0.188, −0.036]	0.004
Male	−0.080 [−0.172, 0.011]	0.085
Female	−0.117 [−0.255, 0.021]	0.097

CI, confidence interval; L/S ratio, liver-to-spleen Hounsfield unit ratio.

**Table A3:** Sensitivity analysis using L1 HU values instead of mean L1–L3 HU values.

Analysis	β [95% CI]	*p* Value
Overall (all ages)	0.082 [0.008, 0.156]	0.030
≤49 years	0.168 [0.040, 0.297]	0.011
50–64 years	0.057 [−0.071, 0.184]	0.384
≥65 years	−0.043 [−0.295, 0.208]	0.733
L/S ratio × age	−0.105 [−0.182, −0.028]	0.008

The overall and age-stratified analyses used L1 HU values as the outcome. The L/S ratio × age row represents the interaction term from the corresponding interaction model. HU, Hounsfield unit; CI, confidence interval; L/S ratio, liver-to-spleen Hounsfield unit ratio.
