# Supplementary material for: Age-Dependent Association of Hepatic Steatosis with Vertebral Trabecular Attenuation in Asymptomatic Adults
Source: J Clin Med. 2026 Oct 28;15(21):8409. doi: 10.3390/jcm15218409 (PMC13764473; doi:10.3390/jcm15218409)
Supplement: Supplementary file 1 [file jcm-15-08409-s001.zip › Supplementary_Material.pdf]

Supplementary Material

Questionnaire on Medical History, Current Conditions, Smoking, and Alcohol Use

1. Medical History and Current Conditions

Q: For each of the following conditions, please indicate your status:

- Currently under outpatient treatment
    - Receiving medication / Under clinical observation (without medication)
- Under observation following a health checkup
- History of treatment
    - Surgery / Medication

Conditions:

1. Hypertension
2. Dyslipidemia
3. Diabetes
4. Cardiac diseases
    a. Angina pectoris / myocardial infarction
    b. Arrhythmia
    c. Other (please specify)
5. Respiratory diseases
    a. Lung cancer
    b. Asthma

c. Tuberculosis

d. Other (please specify)

6. Esophageal, gastric, and duodenal diseases

a. Gastric cancer

b. Gastroesophageal reflux disease (GERD)

c. Polyp

d. Ulcer

e. Other (please specify)

7. Colorectal diseases

a. Colorectal cancer / polyp

b. Inflammatory diseases (ulcerative colitis, Crohn’s disease)

c. Constipation

d. Other (please specify)

8. Liver, gallbladder, and pancreatic diseases

a. Hepatitis B

b. Hepatitis C

c. Liver dysfunction / disorder

d. Fatty liver

e. Cholelithiasis

f. Gallbladder polyp

g. Other (please specify)

9. Hyperuricemia / gout

10. Anemia

11. Kidney and urological diseases

- a. Prostate cancer
- b. Benign prostatic hyperplasia
- c. Renal / urinary tract stones
- d. Chronic kidney disease / renal failure
- e. Other (please specify)

12. Cerebrovascular diseases
    - a. Stroke (cerebral infarction / cerebral hemorrhage)
    - b. Other (please specify)
13. Ophthalmologic diseases
    - a. Glaucoma
    - b. Cataract
    - c. Other (please specify)
14. Allergic diseases (e.g., rhinitis)
15. Rheumatism / collagen diseases
16. Osteoporosis
17. Thyroid diseases
18. Mental disorders / psychiatric diseases
19. Gynecological diseases
    - a. Breast cancer
    - b. Uterine cancer
    - c. Other (please specify)
20. Other diseases (please specify)

2. Tobacco (smoking)

Q: Do you currently smoke?

A: Yes / No / I used to smoke

Q: If you answered "Yes" or "I used to smoke," please provide:

a. Number of cigarettes per day

b. Number of years of smoking

3. Alcohol Consumption

Q: How often do you drink alcoholic beverages (e.g., sake [Japanese rice wine], shochu [Japanese distilled spirit], beer, or Western liquors)?

A: Every day / Occasionally / Rarely

Q: On a drinking day, how much alcohol do you usually consume (converted into sake equivalent)?

A: Less than 1 go / 1–2 go / 2–3 go / 3 go or more

Note: In Japan, the traditional unit "go" is commonly used to measure the amount of sake. One go corresponds to 180 ml of sake and is often used as a standard for comparing other alcoholic beverages.

Reference for 1 go (180 ml of sake):

• Beer (approx. 500 ml)

• Shochu 25% (110 ml)

• Whisky double (1 glass, 60 ml)

• Wine (2 glasses, 240 ml)
